# The Nordic Nutrition Recommendations 2022 – structure and rationale of qualified systematic reviews

**DOI:** 10.29219/fnr.v64.4403

**Published:** 2020-06-18

**Authors:** Erik Kristoffer Arnesen, Jacob Juel Christensen, Rikke Andersen, Hanna Eneroth, Maijaliisa Erkkola, Anne Høyer, Eva Warensjö Lemming, Helle Margrete Meltzer, Þórhallur Ingi Halldórsson, Inga Þórsdóttir, Ursula Schwab, Ellen Trolle, Rune Blomhoff

**Affiliations:** 1 Department of Nutrition, University of Oslo, Oslo, Norway; 2 Norwegian National Advisory Unit on Familial Hypercholesterolemia, Oslo University Hospital, Oslo, Norway; 3 National Food Institute, Technical University of Denmark (DTU), Kgs. Lyngby, Denmark; 4 The Swedish Food Agency, Uppsala, Sweden; 5 Department of Food and Nutrition, University of Helsinki, Helsinki, Finland; 6 The Norwegian Directorate of Health, Oslo, Norway; 7 Department of Environmental Health, Norwegian Institute of Public Health, Oslo, Norway; 8 School of Health Sciences, University of Iceland, Reykjavík, Iceland; 9 Department of Medicine, Endocrinology and Clinical Nutrition, Kuopio University Hospital, Kuopio, Finland, and Institute of Public Health and Clinical Nutrition, University of Eastern Finland, Kuopio Campus, Kuopio, Finland; 10 Division of Cancer Medicine, Oslo University Hospital, Oslo, Norway

**Keywords:** *dietary reference values*, *food-based dietary guidelines*, *systematic reviews*, *national food and health authorities*, *Nordic countries*, *Baltics*, *evidence-based nutrition*, *nutrient recommendations*, *causal relationships*

## Abstract

**Background:**

Qualified systematic reviews (SRs) will form the main basis for evaluating causal effects of nutrients or food groups on health outcomes in the sixth edition of Nordic Nutrition Recommendations to be published in 2022 (NNR2022).

**Objective:**

To describe rationale and structure of SRs used in NNR2022.

**Design:**

The SR methodologies of the previous edition of NNR were used as a starting point. Methodologies of recent SRs commissioned by leading national food and health authorities or international food and health organizations were examined and scrutinized. Methodologies for developing SRs were agreed by the NNR2022 Committee in a consensus-driven process.

**Results:**

Qualified SRs will be developed by a cross-disciplinary group of experts and reported according to the requirements of the EQUATOR network. A number of additional requirements must also be fulfilled, including 1) a clearly stated set of objectives and research questions with pre-defined eligibility criteria for the studies, 2) an explicit, reproducible methodology, 3) a systematic search that attempts to identify all studies that would meet the eligibility criteria, 4) an assessment of the validity of the findings of the included studies through an assessment of ‘risk of bias’ of the studies, 5) a systematic presentation and synthesis of the characteristics and findings of the included studies, and 6) a grading of the overall evidence. The complete definition and requirements of a qualified SR are described.

**Discussion:**

Most SRs published in scientific journals do not fulfill all criteria of the qualified SRs in the NNR2022 project. This article discusses the structure and rationale for requirements of qualified SRs in NNR2022. National food and health authorities have only recently begun to use qualified SRs as a basis for nutrition recommendations.

**Conclusion:**

Qualified SRs will be used to inform dietary reference values (DRVs) and food-based dietary guidelines (FBDGs) in the NNR2022 project.

## Popular scientific summary

- Qualified systematic review (SRs) (i.e. high-quality SRs) are the preferred method for evaluating causal effects of nutrients or food groups on health outcomes in NNR2022.
- Most SRs published in scientific journals do not fulfill all criteria of a qualified SR.
- This article discusses the structure and rationale of qualified SRs in NNR2022.

This article is the second of a three-part series for the Nordic Nutrition Recommendations 2022 (NNR2022):

1. *Principles and methodologies* (1),
2. *Structure and rationale of qualified systematic reviews* (this article), and
3. *Handbook for qualified systematic reviews* (2).

Together, these documents constitute a comprehensive and precise framework for how we plan to update NNR. The first paper (1) describes the organization, principles, methods, and the systematic approach used for the sixth edition of the NNR (NNR2022). The present paper, paper 2, describes aspects of the methodology related to SRs in NNR2022. It provides explanations for the stepwise guidance described in paper 3 (2). The three papers should be considered as a unity.

More than 3 million papers related to diet, food, and/or nutrients have been published in different biomedical scientific journals over the last decades. It is impossible for any individual to keep track of such a constant and enormous production of research results, and studies vary considerably in quality and applicability. A single study is often seemingly overturned by later studies (3). With the evolving and cumulative nature of scientific knowledge, evidence-based practices and guidelines must rely on comprehensive syntheses and critical appraisal of the totality of the evidence. To prevent the choice of evidence from being subjective and skewed (bias), the synthesis should be a systematic, reproducible process, guided by predefined criteria and standards.

Using SRs (Box 1) to develop dietary guidelines is a relatively recent approach, but is increasingly recognized as a crucial component (5, 6). As one of the first nutrition guidelines, NNR implemented an SR approach in the fifth edition, NNR2012. Fifteen *de novo* SRs were developed as part of the NNR2012 project (7).

Box 1 Systematic reviews

A systematic review (SR) approach is used to study the available scientific evidence to allow firm conclusions to be drawn and to minimize influence of reporting bias through comprehensive and reproducible literature searches. In SRs, clearly defined literature search strategies are used together with clearly defined and described selections and reporting protocols to provide a comprehensive and distilled evidence document for the decision makers/working group and to enhance the transparency of the decision-making process (4).

The key characteristics of the SR include:

- A clearly stated set of objectives and research questions with predefined eligibility criteria for the studies (including the outcomes of interest)
- An explicit, reproducible methodology
- A systematic search that attempts to identify all studies that would meet the eligibility criteria
- An assessment of the validity of the findings of the included studies through an assessment of risk of bias of the studies
- A systematic presentation and synthesis of the characteristics and findings of the included studies
- A grading of the overall evidence

According to international standards for guideline development, three principles are directly related to SRs (8):

- Systematic methods should be used to search for evidence.
- The criteria for selecting the evidence should be clearly described.
- The strengths and limitations of the body of evidence should be clearly described.

Still, even among dietary guidelines published after 2010, very few have been based on *de novo* SRs, although some (9–11) did conduct ‘reviews of reviews’, or an umbrella review that is a synthesis of previously published SRs. Very few described methods for identifying the evidence underpinning the guidelines (12).

In addition to the characteristics mentioned in Box 1, a high-quality SR should take into account: 1) research questions addressing specific populations, interventions/exposures and their comparisons, and outcomes; 2) selection and assessment of eligible studies by more than one reviewer; 3) use of appropriate statistical methods if a *meta-analysis* is performed; 4) any heterogeneity across studies; and 5) potential publication/reporting biases (13).

The process, which has been developed by WHO and several other national and international health authorities, can be broadly summarized in five steps (14–19):

1. Formulate research questions and develop a protocol including study eligibility criteria.
2. Systematically search, screen, and select studies for the review according to prespecified eligibility criteria.
3. Extract data from the original studies for analysis, and determine risks of bias in individual studies.
4. Synthesize findings and grade the overall quality/strength of the body of evidence.
5. Make conclusions and report according to reporting guidelines.

A SR may include a quantitative synthesis of research results that estimates an average effect size, a meta-analysis, but always includes a qualitative synthesis. Summarizing effect estimates from several studies yields a more precise estimate. However, the ability of SRs to highlight inconsistencies and/or shortcomings in the body of evidence, as well as its applicability, is just as important for informing decision-makers and recommendations.

Such an approach seeks to curb subjective biases in the selection and evaluation of evidence (although it will likely not be completely eliminated), which in turn strengthens the reliability of the conclusions.

The conclusion of the SRs is not the same as the final recommendations, which are based on several considerations (20). As described in the companion paper (1), there are a number of methodological aspects that uniquely complicate nutrition research. These also have implications for synthesizing and interpreting the nutrition research literature (5, 21–25). Some important specific considerations include the study populations’ nutritional status and background diet; the validity of dietary assessment methods; the bioavailability of nutrients and other food substances; biological interactions of food components; and the many known and unknown factors that may confound or mediate relationships between foods/diets and health outcomes. Comprehensive knowledge of such elements is crucial in the interpretation of nutrition research, in judging the strength of evidence, and finally deriving recommendations.

## Development of guidance for SRs

For NNR2012, a SR methodology was developed based on guidance from the Cochrane Collaboration, the US Agency for Healthcare Research and Quality (AHRQ), and other organizations. With more than 8,000 SRs published yearly (26), SR as a method is an evolving field (27). Thus, the SR methodology developed for NNR2012 has been updated in the NNR2022 project. Some important contributions to this update include the revised edition of the Cochrane Collaboration’s *Handbook for Systematic Reviews of Interventions* and recent updates of AHRQ’s method guides. The Institute of Medicine (IoM) in the United States published the first general standards for SRs in 2011 (14). More recently, there have also been requests for a global harmonization of SR methods specifically regarding nutrition recommendations (28).

The methodology for conducting SRs for NNR2022 is thus primarily based on state-of-the-art recommendations from AHRQ, Cochrane, and IoM, as these are widely used and have a clear theoretical and empirical foundation. Recent principles for dietary reference intakes developed by the National Academies of Science and Medicine (NASEM), the *COSMOS-E: Guidance on conducting SRs and meta-analyses of observational studies of etiology* (29), and the Preferred Reporting Items for SRs and Meta-Analyses (PRISMA), the Meta-analysis of Observational Studies in Epidemiology (MOOSE) and AMSTAR standards for reporting of SRs, were also incorporated. Methods used for SRs in dietary guidelines from other nations and agencies (including USA/Canada, World Cancer Research Fund [WCRF], European Food Safety Authority [EFSA], Australia) were also assessed.

We found that the previous SR methodology guidance for NNR2012 was broadly compatible with more current standards, but a few major changes were needed. Some changes or refinements were made regarding the developing of review questions and protocols, search strategies, and the assessment of risk of bias.

The aim of this paper is to describe the basic structure of the SRs conducted in the field of medical and nutritional sciences, and the rationale underlying each methodological step.**The detailed methodology for SRs performed as part of the NNR2022 project is described in Arnesen et al. (2).

## Organization

The SRs for NNR2022 will be conducted by the NNR-SR Centre consisting of a multidisciplinary group of scientists, at least one statistician and two research librarians. The NNR-SR Centre will be appointed by the NNR 2022 Committee following an open call for experts and a review of their expertise and conflict of interests.

The tasks of the NNR-SR Centre are to develop literature search strategies; search, screen, and select eligible publications for the review; extract data from publications and construct evidence tables; qualitatively and/or quantitatively analyze the evidence; grade the strength of evidence; and write and publish SR reports. The review analysts themselves will not lead the protocol development or eligibility criteria; this will be done by the NNR2022 Committee.

## Identifying and defining research questions and analytic frameworks

A well-formulated review question (or research question) is the backbone of every SR. Usually, several questions are developed. They must be clear and unambiguous and are often stated in a structured format specifying the population, intervention (or exposure), comparator, outcome(s) of interest, timing, setting, and study design (PI/ECOTSS) (Table 1). Not all questions need to include all of these elements, but they will guide the whole process.

**Table 1:** Example of research questions based on the PI/ECOTSS elements (30)

PI/ECOTSS	Example	
Population	Demographics, health status, and so on in the populations receiving an intervention or exposure	Adults
Intervention/exposure	Type of intervention, dosage or level of intake, delivery	Dietary macronutrient composition
Comparator	Placebo/control group, alternative exposure, or other level of exposure	Alternate macronutrient composition
Outcome	Health outcome, surrogate outcome, mortality	Body weight, body fat and/or waist circumference (WC)
Timing	Duration of intervention or follow-up	³1-year follow-up
Setting	Background context, co-intervention, healthcare, and so on	Free-living
Study design	Randomized controlled trial	Randomized controlled trials

An analytic (logical) framework is also useful when developing the review topic and defining eligibility criteria (31). This is an overview of the project and the research question(s), including PI/ECOTSS elements, connecting the intervention/exposure and outcomes. It defines the PI/ECOTSS elements more thoroughly, and illustrates the causal pathways and potential confounding factors to account for. The NNR2022 Committee is responsible for developing analytic frameworks. SR questions will be developed in collaboration between the Committee, the Scientific Advisory Group, and the NNR-SR Centre. Lists of research questions are reported in the protocols (see below) and SR papers.

The PI/ECOTSS form the criteria for including or excluding studies for the review, the *eligibility criteria*. Having prespecified eligibility criteria is one of the defining and essential attributes of SRs, and one of the key factors separating systematic from narrative reviews. Clear, explicit, and predefined eligibility criteria limit the room for subjective and biased selection of studies for the review, and make the review more reproducible (29). Specifically, criteria for types of participants, interventions/exposures, and comparators (for controlled intervention studies), design and time frames, are defined and stated in the protocols. Any changes in eligibility criteria during the search process will be documented in the reviews. However, eligibility criteria will not be changed on the basis of the results.

As the focus of NNR on prevention, studies including only a particular patient group or institutionalized people will often be excluded. Studies including both healthy populations and people with elevated risk of chronic disease or established chronic disease, including obesity, hypercholesterolemia, and hypertension, may be included if deemed appropriate. For intervention studies, criteria for dose level, duration, mode of administration, and so on are also defined.

Eligible study designs are randomized or nonrandomized controlled trials (intervention studies), or observational prospective cohort studies, case-cohort and/or case-control studies. Cross-sectional studies, uncontrolled trials, case reports, and reviews will be excluded. Studies lacking any measure of intake of the foods or nutrients of interest, and studies with multicomponent intervention where the effect of dietary variables cannot be assessed independently, will be excluded.

### Eligibility criteria

For intervention studies, the following criteria are in general predefined. For each specific SR, the criteria may be altered due to certain circumstance. In such cases, it will clearly be stated and the reason for changing the criteria will be described.

- Minimum duration. Should be at least 4 weeks, specific criteria should be defined depending on type of outcome (e.g. risk factor or disease endpoint).

For observational studies, the following criteria are defined:

- Minimum follow-up period. At least 6 months, could be shorter depending on outcome.

For all the study types, the intake ranges for nutrients and dietary sources should be relevant to the Nordic population.

### Protocols

Protocols will be preregistered by the NNR-SR Centre in the PROSPERO (Prospective Register of Ongoing SRs) database (http://www.crd.york.ac.uk/prospero). In reporting the SRs, authors will follow the latest versions of the PRISMA and MOOSE reporting guidelines.

## Literature search

A transparent literature search strategy is one of the major differences between a systematic and a non-systematic (‘narrative’) review. This is essential for minimizing bias in the study selection and for drawing reliable conclusions. There is little direct evidence for how each step in the search process affects the results of the SR, but *not* performing a comprehensive, systematic search comes with large risk (14).

Searching for relevant evidence involves finding a balance between *sensitivity* and *specificity*. The broader (i.e. sensitive) the search strategy, the more evidence will be identified, at the expense of retrieving many non-relevant data as well. The search strategies for the NNR2022 reviews will aim for high sensitivity, using validated search filters by Cochrane and the Scottish Intercollegiate Guidelines Network (www.sign.ac.uk).

Search strategies will be developed for each review question by research librarians or other experts in SRs, in collaboration with the NNR2022 Committee, and build on the PI/ECOTSS components.

Databases searched always include *Medline/PubMed* and the Cochrane Library’s *CENTRAL* register of controlled trials. Review authors may also search *Embase* and/or specialized databases if necessary for the given topic. Reference lists in the included papers and other relevant SRs will also be examined. In addition, cited reference searches – to find newer papers citing the included papers – will be performed with, for example, Web of Science, Scopus, Google Scholar, and so on. Any errata and letters concerning the papers are also examined. The search strategies will be peer reviewed by a research librarian or an expert in SRs outside the review team before the formal search process starts.

Searching for ‘gray literature’, that is, publications outside peer-review journals, such as study protocols, conference abstracts, or government reports, will not be mandatory. Observational studies, which likely will make up a large part of the literature, are rarely preregistered and often do not have a protocol, so the likelihood of finding unpublished studies by searching registries is uncertain (32). Authors of retrieved papers may be contacted for non-reported data or to clarify issues.

No restriction on publication language or date will be included in the search strategy. Both text-word terms and medical subject headings/controlled vocabulary/indexing terms (e.g. MeSH in Medline) will be used.

The methods section in each SR documents the databases and any other sources searched, search dates, and any restrictions. Searches will be updated within 12 months before publication, and then screened for potentially new eligible studies. Dates of the most recent database search are reported in each chapter. Full search strategies will be included as supplementary material accompanying each report.

## Screening and selection of eligible studies

All citations found are screened applying the prespecified eligibility criteria (see above).

This will first be pilot tested with two reviewers independently screening 10% of the titles and abstracts. This will assess the clarity and understanding of the eligibility criteria and the search strategy. If good agreement is not achieved, the eligibility criteria will be refined or clarified (2).

**Fig. 1:**
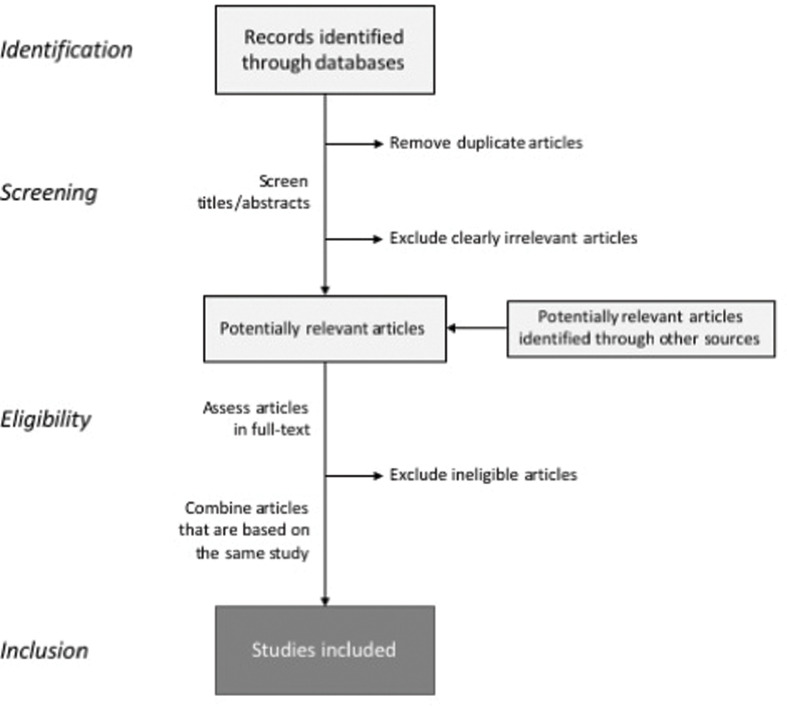
Screening process. Partly adapted from EFSA (19) and Preferred Reporting Items for Systematic Reviews and Meta-Analyses (PRISMA) (33).

At least two or more reviewers will then perform the screening independently. In the screening phase, any disagreements between the screeners will be discussed with a third reviewer to define the discrepancies and make a collaborative decision. First, titles and abstracts of all initially retrieved citations are screened, before full-text publications of those identified as potentially relevant are assessed (Fig. 1).

In the first screening phase, obviously irrelevant articles (based on titles and/or abstracts) will be excluded. If the relevance is uncertain, the article is forwarded to the next step (i.e. an ‘over-inclusive’ approach is used (34)). No data collection or quality assessment will be performed up to this point. If necessary, study authors will be contacted for missing data or other information to clarify eligibility. Masking of study authors, results, and so on will not be performed during the selection process. Citations excluded after full-text assessments will be documented along with reasons for exclusion, and reported in flow diagrams, following the PRISMA standards. Again, reasons for any discrepancies between the reviewers will be discussed and addressed, with inputs from another member of the review team until consensus is achieved.

One study often has multiple reports that can be relevant for the topic. If one study is analyzed in more than one paper, those papers will be linked such that the study itself is the unit.

## Data extraction

The extraction tables developed in this stage will facilitate the later assessment of risk of bias and data synthesis. Qualitative and quantitative data will be collected from each eligible publication and registered in detailed, predefined extraction forms developed for each review. The extraction forms generally include data on source (full reference), eligibility, methodology (e.g. study design, duration/follow-up time, randomization details, blinding), participants and settings, interventions/exposures, endpoints, results (unadjusted and adjusted estimates, except for adjustment models containing variables in the causal pathway), confounding variables and effect modifiers, study sponsor, study author’s conclusions, and reviewers’ comments. Nutrition-specific elements, such as intake levels/dose, food source, method for dietary assessment, validation of dietary assessment method, food composition database used, and assessment of nutrition status, are given a special emphasis (5), and STROBE-NUT guidelines are applied in designing the standardized data extraction form for dietary information (22). As different dietary assessment methods suffer from specific measurement errors, a careful assessment of the method used is essential to allow correct interpretation of the findings.

Data extractions forms will be pretested within the NNR-SR Centre with a handful of included papers to check their feasibility and comprehensibility. The data to be collected, and a procedure to extract the data, will be predetermined by the reviewers, but as for the selection process, data will be extracted by at least two reviewers independently. This is a method for limiting recording errors, which may be large, regardless of the extractors’ experience (35). It is especially important to have two or more persons extracting endpoint data. Again, study authors will be contacted, if necessary, about missing or unclear data.

## Assessment of risk of bias

Before summarizing the research and form conclusions, it is necessary to assess the validity of, or the confidence in, the available evidence. An assessment of risk of bias of the included studies is an important difference between SRs and narrative reviews. This is also a prerequisite for evaluating the overall strength of evidence (see below). Even if the results are consistent across all studies, the conclusions are not sound if the studies are flawed and biased. Importantly, each study must be critically appraised on their own, not simply based on their assigned design labels (such as ‘randomized controlled trials’ [RCTs]) (36).

*Study quality* is a broad concept that includes measurement methods, precision, potential for random errors, applicability, and quality of the reporting (14). Whether the study has a sound research question according to the objectives, and its generalizability, are indicators of *external validity*.

*Risk of bias* is another quality aspect, concerning the study’s *internal validity*. Internal validity refers to whether the study ‘correctly’ answers the research question (29, 37). This affects one’s confidence in the causal effect of the intervention. Compared with the other quality measures, the risk of bias concerns what actually happened in the study, not just how it was designed. A study may be well designed, but still have a high risk of bias.

The Cochrane Collaboration defines *bias* as ‘systematic error, or deviations from the truth, in results or interpretation’, which leads to over- or underestimating the true intervention effect (37). This should not be confused with lack of precision, which leads to *random* errors that can cancel out each other with sufficient replication. Criteria for determining risk of bias in an individual study should be separated from criteria for judging precision, directness, and applicability (36).

Causes of bias are mainly related to the randomization process (in RCTs), deviations from intended interventions, missing outcome data, measurement of the outcome, and selection in the reported result (38). These domains have also been named selection bias, performance bias, attrition bias, detection bias, and reporting bias (see Table 2) (37, 38).

**Table 2:** Sources of bias (37, 38)

Main type of bias	Explanation	Issues
Selection bias – bias arising from the randomization process	Assignment to intervention group is influenced by prognostic factors, leading to systematic differences in background characteristics between the groups being compared (i.e. confounding). Randomized, concealed allocation to groups uniquely limits selection bias.	- Sequence generation (was the recruitment random?)* - Concealed allocation (was the allocation of participants to groups concealed and unpredictable for participants and investigators?)* - Control for confounding factors**
Performance bias – bias due to deviations from intended interventions	Non-protocol interventions given, failure to implement the protocol, or non-adherence to the intervention by participants, due to awareness of intervention assignment.	- Blinding of participants and investigators* - Effect of assignment to intervention (‘intention to treat’) vs. effect of adherence to intervention (‘per protocol’ effect).
Detection bias – bias in measurement of the outcome	Measurement error or misclassification of outcomes. Causes bias if different between the groups.	- Measuring methods appropriate? - Blinding of outcome assessors - Other potential threats to validity, for example, inadequate statistical analyses; exposure assessment method
Attrition bias – bias due to missing outcome data	Systematic differences in attrition or length of follow-up of participants between groups, leading to incomplete outcome data.	- Dropout or loss to follow-up - Missingness is not by chance, but related to intervention group and the value of the outcome
Reporting bias– bias in selection of the reported result	Systematic differences in what outcome measurement or analysis is reported and not.	- Selective endpoint reporting; unfavorable or insignificant findings are less likely to be published (are any prespecified or expected key outcomes not reported?)

* Not applicable to observational studies.

** Applicable to observational studies.

Risk of bias assessment is more complicated with nonrandomized studies, such as prospective cohort studies (32). However, selection bias, performance bias, detection bias, attrition bias, and reporting bias should be assessed (2). Especially, the potential for selection bias is likely higher in most nonrandomized studies, such that the exposed and non-exposed groups are imbalanced when it comes to prognostic factors. It is important to identify these confounding factors and to assess how they were managed.

In observational studies, misclassification (or information/recall) bias may also occur (39), that is, bias due to how the exposure was defined and assessed. There is empirical evidence that several of these risk-of-bias-domains may exaggerate effects of interventions (26, 40). In nutrition, the issue of exposure assessment is especially important to consider when deriving recommendations based on intake-response associations (6, 22, 41).

### Handling of risk of bias in the NNR2022 SRs

The assessment of risk of bias will be planned and described in protocols for each review, where the specific endpoints for which risk of bias assessments to be performed are stated. Risk of bias will be assessed for at least one specific key endpoint for each study by at least two independent reviewers. If disagreements are not resolved by the two reviewers, a third reviewer will be involved. To reduce subjective judgments, the risk of bias assessors are provided with definitions of the domains (i.e. randomization, blinding, attrition, and so on). The response options are ‘Yes/Probably yes’, ‘No/Probably no’, or ‘No information’ (35, 36).

A study may have *low*, *some,* or *high* risk for bias within each domain. The overall risk of bias per study is also judged as *low*, *high,* or *some concerns*. If there is a high risk of bias in one of the mentioned domains, the study is judged to be at high risk of bias overall. A ‘low’ risk of bias judgment does not require that the study is totally free from any type of bias, but that the bias is not so serious that it has any appreciable bearing on the results or conclusions. For instance, blinding is usually not possible in food-based dietary intervention, but this may not always imply a high risk of bias as long as it would not have influenced the outcome. Hence, it is important to acknowledge that the judgment of a study’s risk of bias also depends on to what extent it is likely to affect the results.

There is a lack of strong empirical evidence that one way of assessing the risk of bias is superior. Hence, many different approaches exist (42). The use of quality scales (e.g. the often used *Jadad scale* for trials), in which a summary, numeric quality score is calculated, may be misleading, even causing contradictory conclusions about effects, and is discouraged (29, 42–45). It has become more common to use and classify study quality simply as ‘good’, ‘poor’ or ‘fair’, or similar.

The risk of bias assessments for *de novo* SRs in the NNR2022 project will be based on Cochrane’s risk of bias tool (‘Risk of bias 2.0’) for RCTs (38). For non-randomized trials, the assessments will be based on the recent Risk of Bias in Non-randomized Studies of Interventions (ROBINS-I) instrument, (36, 39) developed by Cochrane collaborators. For observational studies (prospective cohort studies, case-cohort studies or case-control studies), we will use the recently developed ‘Risk of Bias for Nutrition Observational Studies’ (RoB-NObS) tool developed by the USDA’s Nutrition Evidence Systematic Review (NESR) team (46). For each SR, a table/check list with additional questions will be included to add emphasis on bias linked to specific nutrient or diet-related issues such as misclassification due to selection, comparability between exposure groups (i.e. confounding), and exposure and outcome ascertainment (29, 47).

## Data synthesis

After the study data have been extracted and tabulated, and the risk of bias of each study has been assessed, the information will be integrated to allow an interpretation of the overall *body of evidence,* including its quality. The reviewers will qualitatively summarize the findings in summary-of-findings tables. These tables include the main results, including continuous endpoint measures (e.g. mean differences) or categorical endpoint measures (e.g. relative risks or hazard ratios), with 95% confidence intervals. Table 3 is an example of summary table headings.

**Table 3:** Example of a summary table

Exposure/intervention	No. of participants/no. of studies	Outcome variable	Relative risk (RR) (95% confidence interval [CI])	Effect	Risk of bias	Comments	Effect	Risk of bias	Comments
						

There are a number of ways in which studies may be grouped for presentation. Grouping could be done according to study design or other major factors that may influence the results. Forest plots of results can be used to illustrate results of individual studies.

If appropriate, *meta-analyses* will be performed. A meta-analysis is a quantitative combination of study level (or, in some cases, participant level) data from independent studies. Meta-analysis produces an average effect estimate, weighted by study precision. Meta-analysis as a method improves the statistical power to detect differences by increasing the total sample size, providing a more precise effect estimate. It is also useful for highlighting between-study heterogeneity.

However, meta-analyses must be carefully interpreted. They should not always be performed; if the included studies are highly heterogeneous in terms of study populations or settings, interventions and study designs, and/or have low quality, a statistical combination of the data will not give a meaningful answer (48). The meta-analysis may not estimate one ‘true’ intervention effect, but rather a distribution of effects (49). Systematic (i.e. not random) errors from different studies are not cancelled out when studies are combined in a meta-analysis. Thus, when studies show high heterogeneity and/or risk of bias, it is generally discouraged to use meta-analysis. Similar to other types of evidence syntheses, the validity of meta-analyzed results is also affected by the risk of publication or reporting bias – unfavorable or insignificant findings are less likely to be published.

Dose–response effects/relationships will be assessed if sufficient data allow. The meta-analyses will assess statistical heterogeneity between studies, which will be further explored by sensitivity analyses. In the case of considerable inconsistency in results, for example, an *I*^2^ statistic of 75–100% (49), the effect estimates will not be pooled statistically. Sources of heterogeneity to be explored include PI/ECOTSS elements and risk of bias domains. Any sensitivity or subgroup analyses will be prespecified in the protocols of each review. When the risk of bias varies across studies, the primary meta-analyses are restricted to those with low risk of bias, and sensitivity analyses are performed to compare effects according to study quality (50). If 10 or more studies are included in the meta-analysis, tests for publication bias will be performed. Meta-analyses will be performed separately with interventional and observational studies.

## Assessment of the strength of evidence

In the context of SRs, the strength of evidence, or the quality of the body of evidence, refers to the extent one can be confident that the effect estimate across the studies is true (14, 16, 51). The degree of confidence depends broadly speaking on the quality, quantity, and consistency of the evidence base (52, 53).

There is no empirical evidence or agreement on one particular method or terminology for evaluating and describing the quality of the totality of evidence. There are many different systems; a 2005 study identified more than 50 different systems for grading the evidence, and 230 instruments for assessing study quality (14). The most common approach is the *Grading of Recommendations, Assessment, Development, and Evaluation* (GRADE) methodology in which the evidence is categorized as high, moderate, low, and very low quality. Nevertheless, the basic considerations in most evidence quality assessments are:

1. risk of bias (study limitations)
2. consistency of the results (i.e. the relative effect measures)
3. precision (width of the 95% confidence interval around the effect estimate)
4. directness (including external validity)
5. reporting bias (only for RCTs) and, for observational studies, also:
6. dose–response relationship
7. plausible confounding that would have changed the reported effect
8. strength of the association (16, 25, 51).

Evaluation of the strength of evidence for a causal relationship between a food group or a nutrient exposure and a health-related outcome is not trivial. In addition to the many general scientific issues in medical sciences, there are several specific challenges of human nutrition research. The assessment of causality in nutritional studies, and their implication for NNR2022, will be discussed in a forthcoming paper.

### Strength of evidence

In general, the body of evidence for the most relevant outcomes will be evaluated based on all the factors in the GRADE methodology. The approach for grading developed by the WCRF/AICR (23) will be used to describe the strength of evidence. This incorporates the quality and quantity of the totality of the evidence, unexplained heterogeneity in results, the presence of a dose–response relationship, and biological plausibility. This was also used in NNR2012 and previous food-based dietary guideline (FBDG) developments in Norway and Denmark, and in the Global Burden of Disease study (50)

The grading of the evidence for causal relationships, for both reduction and increase in risk, results in one of the following grading categories (Table 4):

**Table 4:** The World Cancer Research Fund (WCRF) categorization of strength of evidence

Evidence of causality	Strength of evidence	Explanation
Convincing	Strong evidence	The evidence is strong enough to judge an association between the exposure and outcome as convincing, and to make a recommendation for reducing risk. This is unlikely to be changed in the light of more studies.
Probable		The evidence is strong enough to support a causal relationship as probable, justifying a recommendation for reducing risk.
Substantial effect on risk unlikely		The evidence is strong enough to support a judgment that there is no substantial causal association between the exposure and the outcome. This is unlikely to be changed in the light of more studies.
Limited – suggestive	Weak evidence	The evidence is too limited to conclude for a probable or convincing causal association but suggests direction of an effect. There may be methodological flaws or few studies, but they show a generally consistent direction. The evidence is rarely sufficient to warrant recommendations.
Limited – no conclusions	Insufficient evidence	The evidence is too limited to make a conclusion. There may be too few studies, too inconsistent directions of effects, or a combination. Most studies have poor quality, or two or more high-quality studies have opposite or negative results. Further research might give evidence for or against a causal relationship with more certainty.

convincing
probable
limited –suggestive
limited – no conclusion
substantial effect unlikely

Grading of ‘*convincing*’ or ‘*probable*’ is generally considered as strong enough evidence for informing dietary reference values (DRVs) and FBDGs (1).

Mendelian randomization (MR) studies may also give support for causal interpretations of observational association, being less affected by confounding and bias than traditional observational studies (1). MR studies have contributed to the establishment of biomarkers such as low-density lipoprotein (LDL)-cholesterol, triglycerides, and blood pressure as causal risk factors for cardiovascular disease, which also have implications for the strength of evidence of nutrition recommendations. Dietary interventions that report sufficiently large changes in such causal risk factors may then be considered clinically relevant and may be more confidently extrapolated to hard endpoints.

In GRADE, RCTs without substantial weaknesses are judged as high-quality evidence, while observational studies *a priori* start with a low-quality grading (but may be upgraded). However, judging RCTs automatically as ‘high-quality’ evidence may be misleading with nutritional interventions due to inherent methodological challenges (6, 54, 55). With the exception of this, the aspects for strength-of-evidence considered by WCRF’s and GRADE’s tools are largely similar in concept. For comparison, GRADE ranks the evidence quality, as shown in Table 5.

**Table 5:** The Grading of Recommendations, Assessment, Development, and Evaluation (GRADE) categorization of strength of evidence

Quality grade	Definition
High	We are very confident that the true effect lies close to that of the estimate of the effect.
Moderate	We are moderately confident in the effect estimate: the true effect is likely to be close to the estimate of the effect, but there is a possibility that it is substantially different.
Low	Our confidence in the effect estimate is limited: the true effect may be substantially different from the estimate of the effect.
Very low	We have very little confidence in the effect estimate: the true effect is likely to be substantially different from the estimate of effect.

With observational evidence, GRADE upgrades the strength of evidence with 1 point if the relative risk (RR) is >2 or <0.5, and with 2 points if RR >5 or <0.2. It is also upgraded by 1 point if there is a dose–response relationship and if any confounding factors would have underestimated the observed effect.

Two reviewers will independently assess each domain and grade the total strength of evidence according to the WCRF criteria in the NNR2022 project. Any disagreement will be solved in a consensus process with the whole group of reviewers.

## Conclusion

SRs will be a major fundament in developing nutrient recommendations or FBDGs in the NNR2022 project. This paper substantiates the specific, stepwise recommendations and expectations of SRs for NNR2022. The methodology for SRs in NNR2022 is informed by current advances in guideline development and SR standards. It could be adapted for use by other countries or organizations formulating nutrient recommendations or FBDGs.

## Conflict of interest and funding

See sections on “Conflicts of interest” and “Sponsors of the NNR2022 project” in the main text of the companion article (1).

## References

[1] Christensen JJ, Arnesen EK, Andersen R, Eneroth H, Erkkola M, Høyer A, et al. The Nordic Nutrition Recommendations 2022 – principles and methodologies. Food Nutr Res 2020. doi: 10.29219/fnr.v64.4402 PMC7307430 32612489

[2] Arnesen EK, Christensen JJ, Andersen R, Eneroth H, Erkkola M, Høyer A, et al. The Nordic Nutrition Recommendations 2022 – handbook for qualified systematic reviews. Food Nutr Res 2020. 10.29219/fnr.v64.4404 PMC7307435 32612492

[3] Ioannidis JP Why most published research findings are false. PLoS Med 2005; 2(8): e124 doi: 10.1371/journal.pmed.0020124 16060722 PMC1182327

[4] Lichtenstein AH, Yetley EA, Lau J Application of systematic review methodology to the field of nutrition: nutritional research series, Vol 1 AHRQ Technical Reviews Rockville, MD: AHRQ; 2009. 20734513

[5] Yetley EA, MacFarlane AJ, Greene-Finestone LS, Garza C, Ard JD, Atkinson SA, et al. Options for basing Dietary Reference Intakes (DRIs) on chronic disease endpoints: report from a joint US-/Canadian-sponsored working group. Am J Clin Nutr 2017; 105(1): 249S–85S. doi: 10.3945/ajcn.116.139097 27927637 PMC5183726

[6] Nordic Council of Ministers Nordic nutrition recommendations 2012: integrating nutrition and physical activity. Copenhagen: Nordic Council of Minsters; 2014.

[7] Chung M, Balk EM, Ip S, Lee J, Terasawa T, Raman G, et al. Systematic review to support the development of nutrient reference intake values: challenges and solutions. Am J Clin Nutr 2010; 92(2): 273–6. doi: 10.3945/ajcn.2009.29092 20504974 PMC2904030

[8] AGREE Next Steps Consortium The Agree II Instrument [Internet]. 2017 Available from: http://www.agreetrust.org. [cited 28 August 2019].

[9] National Nutrition Council Food-based dietary guidelines for public health promotion and prevention of chronic diseases – methodology and scientific evidence [Kostråd for å fremme folkehelsen og forebygge kroniske sykdommer]. Oslo, Norway: Directorate of Health; 2011.

[10] DTU Fødevareinstituttet Evidence-base for the Danish guidelines for diet and physical activity [Evidensgrundlaget for danske råd om kost og fysisk aktivitet]. Søborg, Denmark: Fødevareinstituttet; 2013.

[11] Kromhout D, Spaaij CJ, de Goede J, Weggemans RM The 2015 Dutch food-based dietary guidelines. Eur J Clin Nutr 2016; 70(8): 869–78. doi: 10.1038/ejcn.2016.52 27049034 PMC5399142

[12] Blake P, Durao S, Naude CE, Bero L An analysis of methods used to synthesize evidence and grade recommendations in food-based dietary guidelines. Nutr Rev 2018; 76(4): 290–300. doi: 10.1093/nutrit/nux074 29425371 PMC5914460

[13] Shea BJ, Reeves BC, Wells G, Thuku M, Hamel C, Moran J, et al. AMSTAR 2: a critical appraisal tool for systematic reviews that include randomised or non-randomised studies of healthcare interventions, or both. BMJ 2017; 358: j4008 doi: 10.1136/bmj.j4008 28935701 PMC5833365

[14] Institute Of Medicine Finding what works in health care: standards for systematic reviews. Washington, DC: The National Academies Press; 2011. 24983062

[15] U.S. Dietary Guidelines Advisory Committee Scientific report of the 2015 Dietary Guidelines Advisory Committee: advisory report to the Secretary of Health and Human Services and the Secretary of Agriculture. Washington, DC: United States Department of Agriculture, United States Department of Health and Human Services; 2015.

[16] Schünemann H, Brozek J, Guyatt G, Oxman A GRADE handbook for grading quality of evidence and strength of recommendations. The GRADE Working Group 2013.

[17] AHRQ Methods guide for effectiveness and comparative effectiveness reviews. Rockville, MD: Agency for Healthcare Research and Quality; 2014. 21433403

[18] WHO WHO handbook for guideline development. Geneva: World Health Organization; 2012.

[19] European Food Safety Authority Application of systematic review methodology to food and feed safety assessments to support decision making. EFSA J 2010; 8(6): 1637 doi: 10.2903/j.efsa.2010.1637 PMC13085193 42006960

[20] Brannon PM, Taylor CL, Coates PM Use and applications of systematic reviews in public health nutrition. Annu Rev Nutr 2014; 34: 401–19. doi: 10.1146/annurev-nutr-080508-141240 24819324

[21] Satija A, Yu E, Willett WC, Hu FB Understanding nutritional epidemiology and its role in policy. Adv Nutr 2015; 6(1): 5–18. doi: 10.3945/an.114.007492 25593140 PMC4288279

[22] Lachat C, Hawwash D, Ocke MC, Berg C, Forsum E, Hornell A, et al. Strengthening the reporting of observational studies in epidemiology-nutritional epidemiology (STROBE-nut): an extension of the STROBE statement. PLoS Med 2016; 13(6): e1002036 doi: 10.1371/journal.pmed.1002036 27270749 PMC4896435

[23] Balk EM, Horsley TA, Newberry SJ, Lichtenstein AH, Yetley EA, Schachter HM, et al. A collaborative effort to apply the evidence-based review process to the field of nutrition: challenges, benefits, and lessons learned. Am J Clin Nutr 2007; 85(6): 1448–56. doi: 10.1093/ajcn/85.6.1448 17556679

[24] Willett WC Nutritional epidemiology. 3rd ed. Oxford: Oxford University Press; 2013.

[25] World Cancer Research Fund, American Institute for Cancer Research Continuous update project report. Judging the evidence. London: World Cancer Research Fund; 2018.

[26] Page MJ, Shamseer L, Altman DG, Tetzlaff J, Sampson M, Tricco AC, et al. Epidemiology and reporting characteristics of systematic reviews of biomedical research: a cross-sectional study. PLoS Med 2016; 13(5): e1002028 doi: 10.1371/journal.pmed.1002028 27218655 PMC4878797

[27] National Academies of Sciences, Engineering and Medicine Redesigning the process for establishing the dietary guidelines for Americans. Washington, DC: The National Academies Press; 2017. 29232083

[28] National Academies of Sciences, Engineering and Medicine Global harmonization of methodological approaches to nutrient intake recommendations: proceedings of a workshop. Washington, DC: The National Academies Press; 2018.

[29] Dekkers OM, Vandenbroucke JP, Cevallos M, Renehan AG, Altman DG, Egger M COSMOS-E: Guidance on conducting systematic reviews and meta-analyses of observational studies of etiology. PLoS Med 2019; 16(2): e1002742 doi: 10.1371/journal.pmed.1002742 30789892 PMC6383865

[30] Fogelholm M, Anderssen S, Gunnarsdottir I, Lahti-Koski M Dietary macronutrients and food consumption as determinants of long-term weight change in adult populations: a systematic literature review. Food Nutr Res 2012; 56 doi: 10.3402/fnr.v56i0.19103 PMC3418611 22893781

[31] Helfand M, Balshem H AHRQ series paper 2: principles for developing guidance: AHRQ and the effective health-care program. J Clin Epidemiol 2010; 63(5): 484–90. doi: 10.1016/j.jclinepi.2009.05.005 19716268

[32] Reeves BC, Deeks JJ, Higgins JPT, Wells GA Higgins JPT, Green S Including non-randomized studies. Cochrane handbook for systematic reviews of interventions version 5.1.0. The Cochrane Collaboration; 2011.

[33] Moher D, Liberati A, Tetzlaff J, Altman DG, Group P Preferred reporting items for systematic reviews and meta-analyses: the PRISMA statement. PLoS Med 2009; 6(7): e1000097 doi: 10.1371/journal.pmed.1000097 19621072 PMC2707599

[34] Higgins JPT, Deeks JJ Selecting studies and collecting data. In Higgins JPT, Green S, editors Cochrane handbook for systematic reviews of interventions, version 5.1.0. The Cochrane Collaboration; 2011.

[35] Horton J, Vandermeer B, Hartling L, Tjosvold L, Klassen TP, Buscemi N Systematic review data extraction: cross-sectional study showed that experience did not increase accuracy. J Clin Epidemiol 2010; 63(3): 289–98. doi: 10.1016/j.jclinepi.2009.04.007 19683413

[36] Viswanathan M, Patnode CD, Berkman ND, Bass EB, Chang S, Hartling L, et al. Recommendations for assessing the risk of bias in systematic reviews of health-care interventions. J Clin Epidemiol 2018; 97: 26–34. doi: 10.1016/j.jclinepi.2017.12.004 29248724

[37] Higgins JPT, Altman DG, Sterne JA. Assessing risk of bias in included studies. In: Higgins JPT, Green S, editors Cochrane handbook for systematic reviews of interventions. The Cochrane Collaboration; 2011.

[38] Sterne JA, Savovic J, Page MJ, Elbers RG, Blencowe NS, Boutron I, et al. RoB 2: a revised tool for assessing risk of bias in randomised trials. BMJ 2019; 366: l4898 doi: 10.1136/bmj.l4898 31462531

[39] Sterne JA, Hernan MA, Reeves BC, Savovic J, Berkman ND, Viswanathan M, et al. ROBINS-I: a tool for assessing risk of bias in non-randomised studies of interventions. BMJ 2016; 355: i4919 27733354 10.1136/bmj.i4919 PMC5062054

[40] Page MJ, Higgins JP, Clayton G, Sterne JA, Hrobjartsson A, Savovic J Empirical evidence of study design biases in randomized trials: systematic review of meta-epidemiological studies. PLoS One 2016; 11(7): e0159267 doi: 10.1371/journal.pone.0159267 27398997 PMC4939945

[41] Myers EF, Parrott JS, Splett P, Chung M, Handu D Using risk of bias domains to identify opportunities for improvement in food- and nutrition-related research: an evaluation of research type and design, year of publication, and source of funding. PLoS One 2018; 13(7): e0197425 doi: 10.1371/journal.pone.0197425 29975705 PMC6033375

[42] Wang Z, Taylor K, Allman-Farinelli M, Armstrong B, Askie L, Ghersi D, et al. A systematic review: tools for assessing methodological quality of human observational studies [Draft]: Canberra: NHMRC; 2019.

[43] Herbison P, Hay-Smith J, Gillespie WJ Adjustment of meta-analyses on the basis of quality scores should be abandoned. J Clin Epidemiol 2006; 59(12): 1249–56. doi: 10.1016/j.jclinepi.2006.03.008 17098567

[44] Whiting P, Wolff R, Mallett S, Simera I, Savovic J A proposed framework for developing quality assessment tools. Syst Rev 2017; 6(1): 204 doi: 10.1186/s13643-017-0604-6 29041953 PMC5646161

[45] Juni P, Witschi A, Bloch R, Egger M The hazards of scoring the quality of clinical trials for meta-analysis. JAMA 1999; 282(11): 1054–60. doi: 10.1001/jama.282.11.1054 10493204

[46] Nutrition Evidence Systematic Review. Risk of Bias for Nutrition Observational Studies (RoB-NObs) Tool 2019. Available from: https://nesr.usda.gov/sites/default/files/2019-07/RiskOfBiasForNutritionObservationalStudies-RoB-NObs.pdf [cited 06 February 2020].

[47] National Academies of Sciences, Engineering and Medicine Guiding principles for developing dietary reference intakes based on chronic disease. Washington, DC: National Academies Press; 2017. 29200241

[48] Barnard ND, Willett WC, Ding EL The misuse of meta-analysis in nutrition research. JAMA 2017; 318(15): 1435–6. doi: 10.1001/jama.2017.12083 28975260

[49] Deeks JJ, Higgins JPT, Altman DG Analysing data and undertaking meta-analyses. In Higgins JPT, Altman DG Cochrane handbook for systematic reviews of interventions, version 5.1.0. The Cochrane Collaboration; 2011.

[50] Boutron I, Page MJ, Higgins JPT, Altman DG, Lundh A, Hrobjartsson A Considering bias and conflicts of interest among the included studies. 2019 In: Cochrane handbook for systematic reviews of interventions version 6.0 (updated July 2019) [Internet]. Available from: www.training.cochrane.org/handbook [cited 2019 Oct 17].

[51] Berkman ND, Lohr KN, Ansari MT, Balk EM, Kane R, McDonagh M, et al. Grading the strength of a body of evidence when assessing health care interventions: an EPC update. J Clin Epidemiol 2015; 68(11): 1312–24. doi: 10.1016/j.jclinepi.2014.11.023 25721570

[52] West S, King V, Carey TS, Lohr KN, McKoy N, Sutton SF, et al. Systems to rate the strength of scientific evidence. Evid Rep Technol Assess (Summ). 2002; (47): 1–11. PMC4781591 11979732

[53] Bai A, Shukla VK, Bak G, Wells G Quality assessment tools project report. Ottawa: Canadian Agency for Drugs and Technologies in Health; 2012.

[54] Schwingshackl L, Knuppel S, Schwedhelm C, Hoffmann G, Missbach B, Stelmach-Mardas M, et al. Perspective: NutriGrade: a scoring system to assess and judge the meta-evidence of randomized controlled trials and cohort studies in nutrition research. Adv Nutr 2016; 7(6): 994–1004. doi: 10.3945/an.116.013052 28140319 PMC5105044

[55] Satija A, Stampfer MJ, Rimm EB, Willett W, Hu FB Perspective: are large, simple trials the solution for nutrition research? Adv Nutr 2018; 9(4): 378–87. doi: 10.1093/advances/nmy030 30032229 PMC6054238

